# Climate change drives trait-shifts in coral reef communities

**DOI:** 10.1038/s41598-019-38962-4

**Published:** 2019-03-06

**Authors:** Andreas Kubicek, Broder Breckling, Ove Hoegh-Guldberg, Hauke Reuter

**Affiliations:** 1 Coral Reef Ecosystems Laboratory, School of Biological Sciences, The University of Queensland, St. Lucia, QLD 4072 Australia; 2 Australian Research Council Centre of Excellence for Coral Reef Studies, The University of Queensland, St. Lucia, QLD 4072 Australia; 3 Department Landscape Ecology, University of Vechta, 49364 Vechta, Germany; 4 Global Change Institute, The University of Queensland, St. Lucia, QLD Australia; 5 Department Theoretical Ecology and Modelling, Leibniz Center for Tropical Marine Research (ZMT), 28359 Bremen, Germany; 6 University of Bremen, Faculty of Biology and Chemistry, 28359 Bremen, Germany

## Abstract

Climate change is expected to have profound, partly unforeseeable effects on the composition of functional traits of complex ecosystems, such as coral reefs, and some ecosystem properties are at risk of disappearing. This study applies a novel spatially explicit, individual-based model to explore three critical life history traits of corals: heat tolerance, competitiveness and growth performance under various environmental settings. Building upon these findings, we test the adaptation potential required by a coral community in order to not only survive but also retain its diversity by the end of this century under different IPCC climate scenarios. Even under the most favourable IPCC scenario (Representative Concentration Pathway, RCP 2.6), model results indicate that shifts in the trait space are likely and coral communities will mainly consist of small numbers of temperature-tolerant and fast-growing species. Species composition of coral communities is likely to be determined by heat tolerance, with competitiveness most likely playing a subordinate role. To sustain ~15% of current coral cover under a 2 °C temperature increase by the end of the century (RCP 4.5), coral systems would have to accommodate temperature increases of 0.1–0.15 °C per decade, assuming that periodic extreme thermal events occurred every 8 years. These required adaptation rates are unprecedented and unlikely, given corals’ life-history characteristics.

## Introduction

Ecosystems in all parts of the world are affected by climate change and face increasing risk under future climate projections^1^. Even under the mitigation scenarios agreed on at the Conference of Parties (COP) 21 in Paris in 2015, climate change will cause substantial changes in functional properties of ecosystems and diminished trait diversity in communities^2^. Increasing atmospheric greenhouse gas levels led to a rise in sea surface temperatures (SST) in the Indian Ocean by ~0.65 °C between 1950–2009^3^ and, during the same period, extreme thermal anomalies became more frequent^4^. The first mass coral bleaching events were reported in the late 1970s and these had become a global phenomenon by 1998^5^. Projections based on underlying climate trajectories indicate that both the frequency and intensity of mass coral bleaching events will almost certainly continue to increase^3,4,6,7^.

Scleractinian corals originated in the Middle Triassic and have adapted and evolved to changes in climatic regimes occurring over tens of thousands of years^8^. Today’s rates of climatic change, however, are unprecedented in the past tens of millions of years^1^, meaning that species and communities have to be able to adapt at considerably faster rates. Our simulation approach examines the required rate of adaption by coral species and communities over time but does not discriminate between potential mechanisms such as genetic adaptation, utilization of alternative zooxanthellae, or acclimation^9^. Therefore, we use the term ‘adaptation’ as a general description for a wide set of possible adaptive mechanisms^9–12^.

Population dynamics of scleractinian corals under varying conditions are influenced by life history traits^13^ that, in many cases, are manifestations of distinctly contrary survival and reproduction strategies; i.e. different growth forms (e.g. massive and branching), reproductive modes (brooding/spawning; gonochoric/hermaphroditic), tolerances to stress, and competitive abilities in acquiring and holding space. Physiological processes demand resources and resource limitations give rise to trade-offs among different life history traits, such as competitiveness, stress tolerance and growth rates^14–16^. For corals, a number of studies provide evidence of these trade-offs. Two different patterns of energy allocation for growth, reproduction and lipid storage were identified in a population of *Pocillopora damicornis*^17^; moreover, energy allocation changed in response to tissue damage^18^. In a multi-species empirical study Rachello-Dolmen *et al*.^19^ related life-history traits to environmental conditions. This study found that corallite size influenced stress tolerance, while reproduction and colony growth showed higher levels of constitutive immunity in gonochoric than in hermaphroditic species. In contrast to massive corals which invested most of their resources in immunity, branching species preferentially allocated resources to growth^20^. A modelling study demonstrated how different life history strategies of corals influenced community dynamics under different disturbance conditions: An undisturbed community consisted mainly of massive species, while moderate thermal and mechanical disturbance levels favoured the fast-growing but temperature-sensitive *Acropora muricata*^21^.

Organisms represent combinations of various life-history traits, many of which are affected differently by environmental conditions. Therefore, it is very difficult to identify specific patterns of change in life-history characteristics occurring within a population or community^22^, especially in species rich, complex systems, such as coral reefs^23^. The individual-based modelling approach^24–26^ utilized in this study enables the exploration of the relative influence of different traits and combinations of traits on species development and community composition by providing virtual organisms with specific properties and testing the outcome of their interactions and reactions under different environmental conditions^27,28^. Applying this approach, we can not only assess how different species are likely to respond to future conditions, but also infer how coral life history traits may develop in a future ocean.

Here, we used a spatially explicit individual based model to simulate a coral community which is influenced by the presence of macroalgae, mechanical disturbance events and temperature. We concentrated on 3 important traits in corals: (i) *Growth rate*, which influences the speed at which a coral is able to colonize an area; (ii) *Tolerance to thermal stress*, which reduces the mortality associated with extreme thermal events (i.e. bleaching), and (iii) *Competitiveness with neighbours*, which enables coral colonies to maintain space on a reef and even colonize new ground at the expense of an inferior neighbour when coral cover is high. We populated the model with an array of massive and branching species with different combinations of these three traits, in which pairs of species, one of each morphology, share the same trait combinations (see Methods: Simulations; Appendix, Table S1). To represent the respective position of each species in trait space we apply the triangle approach originally developed by Grime^29^ for terrestrial plants (Appendix 1: Fig. S2). Each array consisted of 20 species and each simulation run consisted of multiple replications of 10 different initial coral community distributions, whose results were combined for analysis. Thus the initial number of species in each simulated scenario was 200 species. Community dynamics were simulated over 90 years. We used these model simulations to explore the influence of key environmental parameters on coral cover and community composition (Appendix 1: Table S2). Furthermore, we set out to identify the trait combination(s) that will enable coral species of each growth form to be ‘successful’ (‘winners’ *sensu* Loya *et al*.^30^) under future temperature conditions predicted by climate change scenarios.

In Scenario 1 (used as a baseline) we analysed how initial coral cover and the occurrence of mechanical disturbance events influence a coral community that is not stressed by coral bleaching. In Scenario 2 we added different frequencies of extreme thermal events to explore the recovery potential of coral communities and trait-based changes in community composition following bleaching events. Scenario 3 extends on Scenario 2 by adding gradual increases in temperatures, equivalent to temperature increases under different IPCC Representative Concentration Pathways, i.e. RCP 2.6 to 8.5, but with constant rates of change^1^. In the latter scenario we also identified adaptation rates required to enable coral communities to sustain themselves under different climate change scenarios, by comparing the responses of fast-adapting and more slowly adapting coral communities. Differences in adaptation rates were mimicked in the model by calculating degree heating days (DHD) based on deviations from long-term mean summer temperatures (LMST) calculated over different time periods (10 to 50 years; see Methods). The use of shorter time period to calculate LMST results in lower DHD values and thus reduced bleaching susceptibility in the coral community. Adaptation rates are expressed as degrees Celsius per decade. These values represent the seawater temperature increase that coral species would need to accommodate in order to survive.

## Results

### Scenario 1: Base line scenario - constant temperatures without bleaching

**Figure 1:**
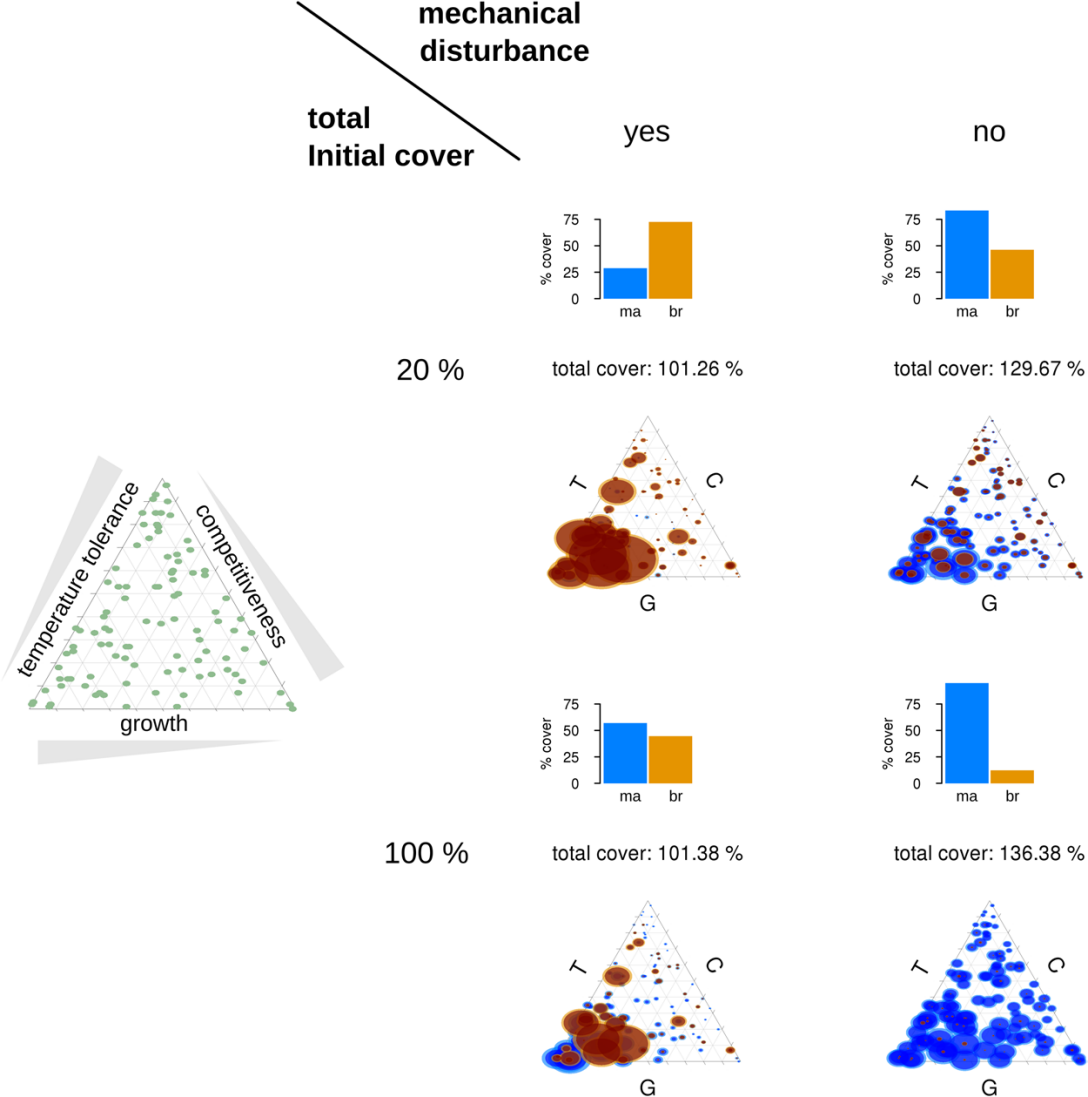
Scenario 1: The coral community after 90 years of simulation time without extreme thermal events. The left-hand triangle shows show the initial coral community and the distribution of specific trait combinations (Temperature tolerance, Competitiveness, and Growth rates). Within the main plot, simulation results are shown with a total initial coral cover of 20% (upper charts) and 100% (lower charts), with (left) and without (right) the occurrence of mechanical disturbance events. The resulting percentage cover of massive (‘ma’, blue) and branching (‘br’, orange) coral species is represented with bar charts above the respective triangle plot. In the trait space a circle centre indicates the trait combination for a species and the circle size indicates the coverage (mean in dark ± SD in lighter colours) of this trait combination.

Direct competition between coral species played an important role when simulated reefs were undisturbed by extreme thermal events and mechanical disturbance events (Fig. 1). In these conditions trait diversity within the community stayed high (Appendix 1: Table S3) and nearly all species survived. The occurrence of mechanical disturbance events favoured branching corals when total initial coral cover was low (20%), but not when initial cover was high (100%). Furthermore, the occurrence of mechanical disturbance levels favoured fast-growing coral species over competitive ones.

### Scenario 2: Survival after 90 years with a constant frequency of extreme temperatures

**Figure 2:**
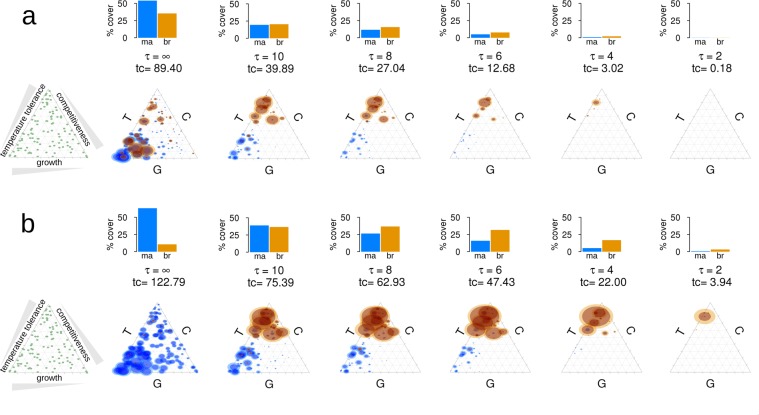
Scenario 2: The surviving coral community after 90 years with respect to species trait combinations (**a**) with and (**b**) without the occurrence of mechanical disturbance events. The plots on the far left show the initial trait compositions. The plot second to the left shows the results when no extreme thermal events occurred during the simulation time (where the interval between bleaching events, τ, equals infinity). In the following plots the frequency of extreme thermal events increases from left to right (with values of τ decreasing from 10 to 2 years). Bar charts indicate the percentage cover of massive (‘ma’, blue) and branching (‘br’, orange) corals, and ‘tc’ indicates total coral cover. In the triangle plots below the bar charts the center of a circle indicates the specific trait combination and the size of the circle indicates the relative contribution of a species to the whole community’s covered area.

In the absence of extreme thermal events and mechanical disturbances, the trait diversity within the coral community remained high (Fig. 2b) and massive corals dominated over branching ones by a ratio of 10:1. In the absence of extreme thermal events, mechanical disturbance provided relatively favourable conditions for branching corals (Fig. 2a). Species diversity decreased with increasing frequencies of extreme thermal events (Appendix 1: Table S4). This effect was more pronounced when mechanical disturbance also occurred (Fig. 2a,b). At the same time there was a shift to fast-growing species in massive corals and temperature-resistant species in branching corals, at the expense of species which mainly invested in competitive traits.

### Scenario 3: Gradually increasing temperatures

Results for Scenario 3 are given for fast-adapting communities which were able to adapt within 10 years to warming sea temperatures and for slow-adapting communities which required up to 50 years.

#### No extreme thermal events during simulations

**Figure 3:**
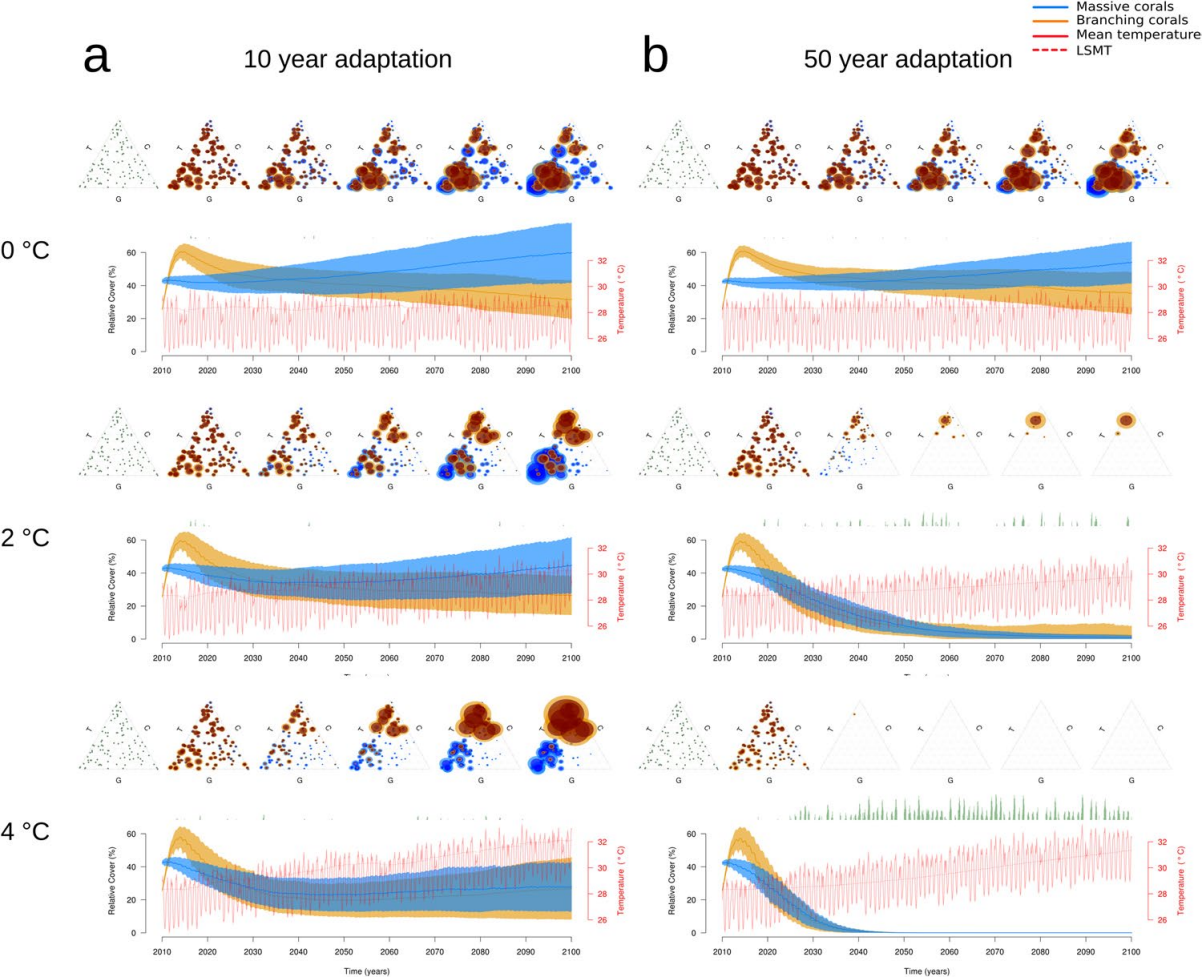
Scenario 3: The effect of gradually increasing temperatures on (**a**) fast-adapting coral communities (*LMST* calculated using a 10-year base period) and (**b**) slow-adapting communities (*LMST* calculated using a 50-year base period), if extreme thermal events would not occur. The graphs show the relative cover (mean ± SD) for massive (blue) and branching (orange) corals over time for a temperature increase by 2 and 4 °C over 90 years. The triangle plots above show the initial trait compositions (far left triangles), and their evolution for both morphotypes within the coral community in 20-year steps, starting from 2020 (i.e. 10 years after the start of the simulation). In the trait space a circle centre indicates the trait combination for a species and the circle size indicates the coverage (mean in dark ± SD in lighter colours) of this trait combination. Monthly mean temperatures are indicated in red solid lines and long-term summertime mean temperatures (*LMST*) in red dashed line. The green bars indicate the temporal occurrence and the length of a bar indicates the magnitude (degree heating days) of extreme thermal events.

**Figure 4:**
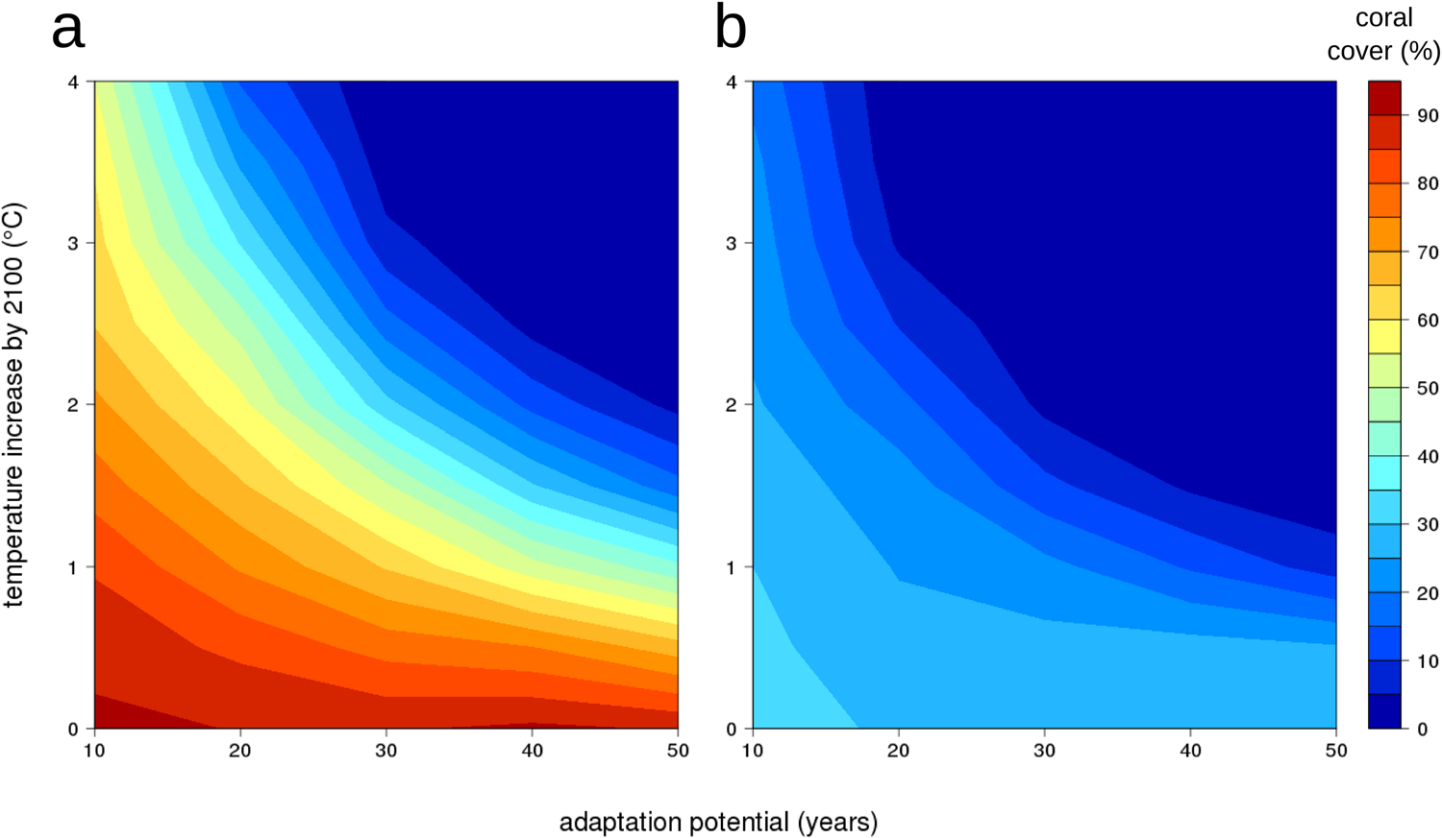
Scenario 3: The relative coral cover in the year 2100 under different scenarios of gradual temperature increase against different coral system adaptation potentials without extreme thermal events (left) and with extreme thermal events occurring every 8 years (right).

Even simulated temperature increases of 4 °C (RCP 8.5) above current conditions did not lead to mass coral bleaching in fast-adapting coral communities in the absence of extreme thermal events (Figs 3a and 4a). Coral cover was ~60% of initial cover and an average of 90 of the initial 200 species survived (H = 3.81). Nevertheless, with faster temperature increases, community composition shifted from fast-growing to more heat-tolerant species.

On the other hand, coral cover declined tremendously for slow-adapting communities (Fig. 3b). Half of the coral cover was lost with a 1 °C temperature increase (RCP 2.6) and, with only a third of the species surviving, diversity was lower than for fast-adapting communities under highest simulated temperature increase of 4 °C. By the end of the simulation these communities mainly consisted of heat-tolerant branching species and to a lesser extent of fast-growing and heat-tolerant massive species. At a temperature increase of 2 °C (RCP 4.5) only 5 heat-tolerant species of 200 initial species survived for the entire simulation period of 90 years. The amount and magnitude of bleaching events steadily increased over the course of the simulation and after 70 years all massive species were extinct. If the temperature increased ≥3 °C, bleaching and mortality events became so frequent and strong that none of the coral species survived longer than 50–60 years.

In the absence of extreme thermal events corals would have to adapt by about 0.06 °C per decade to sustain approximately 20% of the initial coral cover with a temperature increase of about 2 °C by the end of the century (Fig. 4a).

#### Extreme thermal events occurring every 8 years

Here, we found that the potential adaptation rate needed to be 0.2 to 0.22 °C per decade to allow for survival of ~20% of initial coral cover by the end of the century with a 2 °C (RCP 4.5) temperature increase (Fig. 4b). To survive a 4 °C temperature increase the community would have to adapt at about 0.45 °C per decade to sustain itself. For fast-adapting communities, the magnitude of extreme thermal events was buffered. Therefore, even with a 4 °C temperature increase, fast-adapting communities still maintained 20% of initial cover, of which heat-tolerant branching species covered ~15% and fast growing massive species ~5% (Appendix 1: Fig. S1). In slow-adapting communities, only a few heat-tolerant branching species survived a temperature increase of 1 °C.

## Discussion

Our results demonstrate that only specific life-history traits enable corals to persist under conditions of a global increase in seawater temperature and, possibly, contribute to community recovery under more stable climatic conditions in the future. Nevertheless, as many corals are already living at their physiological limits^5,31,32^ and given their long, sometimes multi-decadal generation times^33^, it is unlikely that the speed of adaptation can keep up with the pace of climate change.

Direct competition among coral species only played an important role when simulated reefs were left undisturbed by extreme thermal events and mechanical disturbance events (Fig. 1, Appendix 1: Table S3). Branching corals were favoured by the occurrence of mechanical disturbance events, as well as by low initial coral cover (in Scenario 1). Disturbances create recruitment opportunities for fast-growing branching or stress-tolerant coral species, irrespective of their type, which may then even form canopies that shade out other organisms or prevent them from settling^34,35^. Such dynamic environments considerably decrease the importance of direct competition with neighbouring organisms^34,35^ (Fig. 1). However, in a trade-off, fast-growing branching species (e.g. *Acropora*) are very often more sensitive to bleaching. Consequently, under climate change conditions the dominance of these species might increase ecosystem resilience only in the mid-term, because they exclude more heat-tolerant species, which could subsequently result in the eradication of entire reef communities affected by recurring extreme thermal events^16^.

If extreme temperature events occurred at intervals of 10 or fewer years, species composition in branching corals underwent a clear shift to heat-tolerant species (Fig. 2). In massive corals, fast-growing and heat-tolerant species dominated. Populations of heat-tolerant species can survive periods of scarce recruitment^36^ and, once temperature-sensitive competitors were eliminated, more heat-tolerant species were able to increase population sizes and hence their reproductive output for future generations. This effect has also been observed in the putative shift from branching to massive growth forms in outer reef slope communities in Moorea, French Polynesia^37^.

The adaptation potential of the coral community will be of central importance for the future of reefs and has to be treated as a crucial parameter in predictive coral reef models^10^. In our simulations we found that fast community adaptation rates (up to 0.45 °C per decade) prevented bleaching almost entirely for moderate thermal events and buffered the effects of extreme thermal events. Even temperature increases of 4 °C (RCP 8.5) by the end of the century (Fig. 3a) affected these communities only marginally. Other studies in which the authors use a similar rolling climatology to analyze the adaptation potential of coral reefs report similar results, and conclude that coral community adaptation rates of up to 0.4 °C per decade^10^, or 0.2–1 °C per decade^38^ are required in order for coral reefs to survive to the end of this century. Our results show clearly that using an overly short base period for the calculation of the *LMST* can lead to an underestimation of the severity of future bleaching events (Fig. 4, Appendix 1: Fig. S1), and hence, an overestimation of coral survival. When the model incorporated slower adaptation rates (i.e. 0.022–0.09 °C per decade, corresponding to the calculation of *LMST* over 50 years), there was a strong decline in coral cover and mainly temperature-tolerant branching species survived to the end of the simulation period. Massive species were selected for fast growth and heat tolerance which also happened at Sesoko Island reefs, Japan after a bleaching event^39^. In our study, while trajectories in total cover behaved similarly for massive and branching corals (Fig. 3, Appendix 1: Fig. S1), the underlying mechanisms were substantially different. Compared to branching corals, massive corals generally suffered less from mechanical or thermal disturbances, but recovered at low rates due to slower growth. Branching corals were more sensitive to both modes of disturbance and their populations always suffered high disturbance mortality. Nevertheless, due to their fast growth rates they were able replenish losses from disturbances in a few years.

Global temperatures will rise 1–4 °C above current conditions^1^ by 2100, depending on the effectiveness of collective action to deal with climate change. Our results indicate that some coral species have a good chance of survival under the RCP 2.6 scenario (~1 °C above current conditions; 1.5–2.0 °C above pre-industrial temperature levels) if extreme thermal events do not occur more frequently than every 8 years. This estimation may be considered optimistic in the light of the 5 year return period of extreme thermal events suggested by Frieler *et al*.^40^, the effects of two major bleaching events observed recently^41^, and results of other studies indicating a high likelihood of increased frequency and intensity of extreme thermal events^4–6^. All other IPCC scenarios, with temperature increases of 2 °C (RCP 4.5) and above (RCP 6.0 and RCP 8.5) will almost certainly have devastating impacts, as previously demonstrated^5,10,38,39^. According to our results, coral communities would be strongly selected for heat tolerance and only marginally for fast growth.

The rates at which corals can adapt to increasing temperatures and increased frequency of extreme thermal events is the subject of controversy, which is unsurprising given the many different potential adaptation mechanisms involved. These may include genetic and epigenetic adaptation of the coral^42–44^, the influence of the associated microbiome^45^, hybridization between similar species^46–48^, or somatic mutations^49,50^, which may even be inheritable^51^. Symbionts can significantly influence adaptive traits of corals^32,52^. Within the holobiont the compostition of hosted zooxanthellae can shift to heat tolerant species (symbiont shuffling)^53^ or the holobiont may switch to heat tolerant *Symbiodinium* strains after bleaching (symbiont switching)^54^, both of which may reduce fitness and/or growth^55,56^. Some models incorporate symbiont evolution^57^ or genomic adaptation^58^, which improves coral survival significantly for moderate thermal events and may secure the survival of some species over the next 100–250 years^59^.

This simulation study explores life-history traits and interactions in coral reefs with an unprecedented level of detail and allows new insights into how coral communities cope with different climate change scenarios. Nevertheless, as with every model, the results have to be evaluated in context of the underlying assumptions and constraints. Within this study all species had the same amount of resources to allocate to traits (there were no super-organisms) and we only allowed the species to allocate resources once, in order to extrapolate which trait combination would be best fitted to specific environmental conditions. Temperature adaptation was mimicked on the community level, but we did not allow for adaptation or evolution of traits for single species during simulation runs, which might alter long-term outcomes significantly. We took account of mechanical disturbance events and temperature induced bleaching of corals but left out the effect of increased CO_2_ concentrations in the sea water, which has been shown to have an effect on calcification and other physiological properties of reef organisms^60,61^. The introduction of new traits or species due to migration between reefs, as well as the succession on a potentially viable bare area were not considered in this study, but are topics which could be addressed using the same modelling approach in future investigations.

We kept the biological ranges of coral traits in a moderate range to explore the fate of existing coral communities in a future climate. Furthermore, our study explored a theoretical trait-space for coral species and did not aim to extrapolate these simulations on a global scale. This would require identification of representative reefs worldwide and, for each reef, a description of the combinations of traits and species present, current environmental conditions and local SST projections, as demonstrated by Logan *et al*.^10^.

Based on analysis of the limited—albeit most relevant—set of factors incorporated in our model, the results highlight that radical measures have to be taken to preserve even a fraction of present-day coral abundance and diversity. Without rapid adaptation, coral communities will undergo a severe decline in biodiversity, functional diversity, and species abundances despite all management efforts^62^. Achieving stability in the earth’s climatic conditions is vitally important if we are to preserve coral reefs which are among the most vulnerable ecosystems on this planet.

## Methods

We used an individual-based model to simulate a benthic coral reef community with massive and branching coral species and algae. To test the influence of changing environmental conditions we utilized various scenarios in which different coral species and algae compete for space on a two-dimensional, continuous simulation area (50 × 50 m). The substrate was defined as suitable for the indiscriminate settlement of corals and algae. Organisms within this space grew every month, dynamically interacting with neighbouring organisms and their environment. The community is exposed to thermal events which can induce bleaching in individual coral colonies, as well as mechanical disturbance events which remove affected colonies from the simulation area. The model was programmed in Java and the analysis of results was conducted with R Statistical Software. The source code is available on GitHub (https://github.com/danukub/siccom_v3).

### Corals

In our model, coral colonies were individual entities that grew, reproduced, and interacted with their direct neighbours. Each of these activities was influenced by environmental conditions. The characteristics of the corals were representative of massive and branching morphologies which differed in certain life history traits (Appendix 1: Table S3). The surface factor (as explained below) was deemed to be higher for branching corals and ultimately determined growth rates and the ability of corals to reproduce by fragmentation.

### Implementation of random trait combinations

An array of corals species with different combinations of the three traits (i) growth rate, (ii) susceptibility to bleaching (i.e. heat-tolerance) and (iii) competitiveness was generated at the beginning of each simulation. Each coral species was given a total of 100 resource units (RUs) which were randomly distributed to specify the strength of the three traits (i.e. a species could maximize the capacity of one trait or of allocate resources in different combinations and proportions of either two or all three traits). Coral species in the model can thus be subdivided into 4 groups; i.e. (a) Heat tolerant, (b) highly competitive, (c) fast growing, and (d) those with balanced (mixed) traits (Appendix 1: Fig. S2).

### Surface factor

The surface factor determines the compactness of the coral. We assume a hemispherical surface area for massive corals which is then multiplied by the surface factor to account for three-dimensional structure of branching corals (see Table S1 for values). The surface factor was theoretically set to achieve a 3-D structure comparable to rugosity measurements in coral reefs and to account for differences between massive and branching corals^21^. In turn, the surface factor determines (i) the lateral extension rate, as a compact colony skeleton structure does not extend into space as fast as a branching one, (ii) the probability of being removed during a mechanical disturbance event, and (iii) the probability of producing fragments that may subsequently settle and grow into autonomous colonies.

### Growth and competition

Each individual coral colony within the simulation grows with a species-specific growth rate, corresponding to the trait combinations, as described above (Table S1). As it has been shown that competition among corals affects growth rates of competitors^63,64^, the outcome of direct competition between neighbouring coral colonies is determined using a competition index (*CI*, similar to Langmead and Sheppard)^65^, which influences directional growth of affected colonies at each model step (see Appendix 1: Method section).

### Coral Bleaching

Mass coral bleaching and mortality due to thermal stress is a consequence of the frequency and intensity of temperature anomalies^60^. We used a combination of degree heating days (*DHD*) and heating rates (*HR*) to project bleaching events^66^. For most simulations, the long-term mean summer temperature (*LMST*) was calculated from the warmest three months of the last 50 simulation years and updated monthly. To calculate *DHD* for a month we summed up positive differences between daily temperatures and *LMST* for the last 120 days. If 40 *DHD* were exceeded in these last 120 days, we calculated the *HR* for this month, by dividing *DHD* by the number of days where temperatures were higher than *LMST* to determine the *HR*. The maximum heat rate (*HR*_*max*_) of 3.5, i.e. the *HR* at which all corals bleach and die, was defined according to Maynard *et al*.^66^. From this value we estimated a minimum heat rate (*HR*_*min*_) and specific minimum bleaching probabilities (*bp*_*min*_) to fit the bleaching and mortality index for the temperature data of the year 1998 combined with a long-term climatology from Zanzibar Island (see below) and data for the 1998 bleaching event in Kenya^67^. The actual bleaching probability (*bp*_*actual*_) was then determined based on the *HR* for each month (see Appendix 1: Methods section). Corals differ in susceptibility and bleaching mortality according to their morphology, with some massive coral species being slightly more tolerant to higher temperatures^30,37,67^.

### Adaptation potential of the coral community

A crucial factor for determining *DHD* and *HR* is the number of years from which *LMST* is calculated. In the original method a base period of 11 years was used to calculate *LMST*^66^. If *LMST* is calculated using a rolling climatology, the period of time that is used for the calculation of *LMST* is also the system’s response time in adapting to changing temperature dynamics^10,68^. In biological terms, using fewer years to calculate *LMST* mimics a faster adaptation rate of coral communities; i.e. assuming overall increasing temperatures with a shorter base for calculating LMST the difference between LMST and actual daily temperatures, and hence the values for DHD and HR decrease. In this study, we calculated *LMST* using base periods ranging from 10 to 50 years in order to estimate the consequences of different adaptation rates for coral communities.

### Algae

Macroalgae were implemented as individuals, which occupy space and can displace coral recruits ≤4 months old. Macroalgae occupied free space fastest, but were then slowly displaced by corals (see also Appendix: Macroalgae and coral-algae interactions). In simulations we used algae as coral competitors, but did not evaluate their influence on coral performance, as they affected all species equally.

### Environmental conditions

The climatology (1997–2010) used in the present study originates from Chumbe Island, Zanzibar (C. Muhando, Centre for Marine Science, Zanzibar, unpublished data). This time series includes the 1998 El Niño mass bleaching event, when water temperatures approximately 2 °C above the long-term average (in March) caused severe coral bleaching and mortality across the Western Indian Ocean. To simulate longer time spans, single years of the data set were concatenated in a random order. Temperature data for 1998 was treated differently, as the high temperatures for this year were an extreme thermal event. These data were excluded from the main climatology and used to simulate the occurrence of extreme thermal events, which were inserted as a fully controlled component of the scenarios (see below). Disturbance events were included as an external mechanical impact, which killed all organisms in a disturbed area, and thus created space for settlement. In the scenarios mechanical disturbance events occurred either (i) never, or (ii) as smaller (2–4 m in diameter) and larger (5–10 m in diameter) events, which occurred every 6 and 12 months, respectively.

### Simulations

We assessed how environmental conditions determined survival of traits and shaped coral communities within the simulation environment (Table S2). The baseline scenario (Scenario 1) explored conditions similar to those of previous decades when thermal stress did not play a big role in the community dynamics of coral reefs. In order to understand the impact of mass coral bleaching and mortality, this baseline scenario was compared to those where thermal stress was significant (Scenarios 2–3). In each simulation run a total of 20 coral species (10 massive and 10 branching) were included with pairs of species (massive and branching) sharing the same combination of traits in relative terms; absolute values for traits differ between massive and branching species in each trait combination pair (see Appendix 1: Table S1). For each scenario setting we simulated 10 different combinations of species (amounting to 200 species in total) and ran 20 replicates for each of these combinations of species with varying initial trait combinations.

Randomly sized coral colonies of all species were randomly distributed over the simulation area at the beginning of a simulation. Individual colonies can be overgrown to a certain extent, so that total coral cover may exceed 100%, but overgrowing branching corals are displaced if area overlap is too large. At the start of the simulations, massive corals outcompeted branching corals that occupied the same space; thus coral cover of massive species was higher in initial stages of the simulations. Over time, this effect was compensated by higher growth rates of branching corals, which grew into free space after disturbance events.

Community dynamics were simulated over 90 years. Based on the situation at the end of 90 years, means and standard deviations of relative coral cover were calculated for each species, and community diversity (Shannon index) and evenness were assessed for each scenario.

## Scenarios

### Scenario 1: Base line scenario - constant temperatures without bleaching

In these scenarios we assessed the competition between neighbouring coral colonies with and without mechanical disturbance events. The simulation temperature scheme was kept on low oscillations without extreme thermal events, so no bleaching occurred throughout the simulation runs. To analyse the influence of space availability, we ran scenarios with initial total coral cover of 20% and 100%, i.e. with each species occupying 1% and 5% of the total area, respectively.

### Scenario 2: Survival after 90 years with a constant frequency of extreme temperatures

In order to analyse trait performance within a stressed community, we simulated reef conditions with extreme thermal events (i.e. El Niño years) recurring at a constant interval of 2, 4, 6, 8 or 10 years, or never in the control scenario. For Scenario 2, initial coral cover was 100% and each species initially covered 5% of the simulation area.

### Scenario 3: Gradually increasing temperatures

In this scenario sea temperature was gradually increased (in 0.5 °C increments) to reach 0.5–4 °C above current conditions by the end of this century. This was achieved by adding the respective increment to the generated temperature scenarios, thus keeping the original variability of the climatology. An increase of 0.5–1.5 °C corresponds to RCP 2.6 for the Indian Ocean, while an increase of 2.5–4 °C corresponds to RCP 8.5^1,3^. In these scenarios, we also analysed the influence of the adaptation potential of coral species by simulating different response times (10 to 50 years) to changing temperature dynamics (i.e. mimicking fast adaptation by using *LMST* based on 10 preceding years, and a slower response using LMST for a 50 year period). In all cases, initial coral cover was 100% and each species initially covered 5% of the simulation area. All scenario runs were assessed with and without the effect of recurring extreme thermal events in 8 year intervals, which is in the range of estimations for current intervals of extreme thermal events^4,40^.

## Supplementary information

Supporting Information
