# Supplementary material for: Climate change drives trait-shifts in coral reef communities
Source: Sci Rep. 2019 Mar 6;9:3721. doi: 10.1038/s41598-019-38962-4 (PMC6403357; doi:10.1038/s41598-019-38962-4)
Supplement: Supplementary file 1 — Supporting Information [file 41598_2019_38962_MOESM1_ESM.pdf]

Supporting Information

Climate change drives trait-shifts in coral reef communities

Andreas Kubicek, Broder Breckling, Ove Hoegh-Guldberg, Hauke Reuter

Table S1 Different traits and their parameter settings. Growth rates for representative species were taken from [1-27] (see also [28], Table 1)

Trait	Levels		Mode	Comments
**Growth form**	Massive	Branching	discrete	
**Surface factor**	1 – 1.5	3 – 5	continuous	Determines the structural complexity of a coral species colony
**Growth rate (mm)**	5 – 30		continuous	Determines the growth rate for a coral species in millimeters when multiplied by the surface factor.
**Reproductive output**	1300		constant	Determines the reproductive output per square cm of a coral species; divided by the surface factor
**Retention rate**	0.00000001 (1 * 10E-8)		constant	Determines the fraction of recruits which will stay on the patch after spawning
**Aggressiveness in direct competition (*CI*)**	1 – 10		continuous	Determines the success when a colony is in direct competition with other organisms
**Minimum bleaching probability**	0.1 – 0.3	0.1 – 0.45	continuous	Determines the bleaching probability at specific temperatures
**Minimum bleaching mortality**	0.1 – 0.2	0.1 – 0.3	continuous	Determines the probability of dying while bleached for specific temperatures

Table S2 Overview of the environmental settings used in the different scenarios.

Scenario	Thermal effects	Mechanical disturbances
1 Baseline	-	yes/no
2 Constant thermal stress	intensity: major bleaching event<br>frequency: every 2-10 years, or never	yes/no
3 Increasing thermal stress	increasing temperatures<br>major bleaching events occurred never or every 8 years, respectively	yes

Results

Table S3 Shannon Indices, evenness, and species richness after 90 years of simulation time without any bleaching events.

Total initial cover	Mechanical disturbances	H index	Evenness	*n* Species
20	no	5.10	0.71	200
	yes	4.40	0.64	149
100	no	4.74	0.66	156
	yes	4.60	0.67	157

Table S4 Shannon indices, evenness and species richness for varying frequencies of major bleaching events.

Disturbance events	Bleaching frequency	H-Index	Evenness	*n* Species
No	∞	4.69	0.66	137
	10	4.23	0.64	116
	8	3.93	0.61	98
	6	3.43	0.56	66
	4	2.57	0.48	29
	2	0.97	0.28	6
Yes	∞	4.58	0.68	149
	10	3.84	0.65	75
	8	3.40	0.62	50
	6	2.71	0.59	25
	4	1.42	0.46	6
	2	0.00	0.00	0

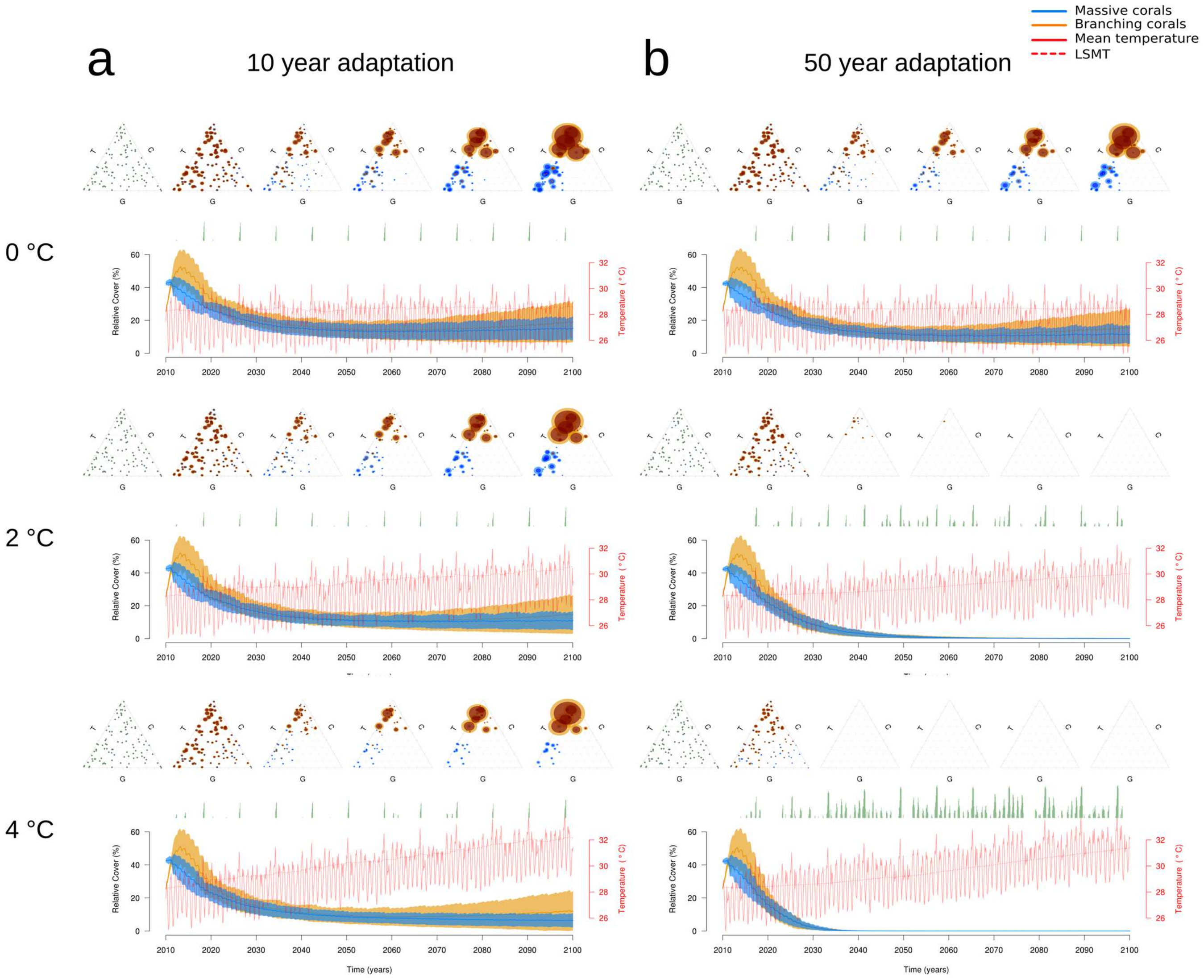

Figure S1, Scenario 3: The effect of gradually increasing temperatures on (a) fast-adapting coral communities (*LMST* calculated using a 10-year base period) and slow-adapting communities (*LMST* calculated using a 50-year base period), if major bleaching events would occur every 8 years. The graphs show the relative cover (mean ± SD) for massive (blue) and branching (orange) corals over time for a temperature increase by 1-4 °C over 90 years. The triangle plots above show the trait composition within the coral community for both morphotypes in 20-year steps, starting from 2020 (i.e. 10 years after the start of the simulation). In the trait space a circle centre indicates the trait combination for a species and the circle size indicates the coverage (mean in dark ± SD in lighter colours) of this trait combination. Monthly mean temperatures are indicated in red solid lines and long-term summertime mean temperatures (*LMST*) in red dashed line. The green bars indicate the temporal occurrence and the length of a bar indicates the magnitude (degree heating days) of extreme thermal events.

Methods and model implementations

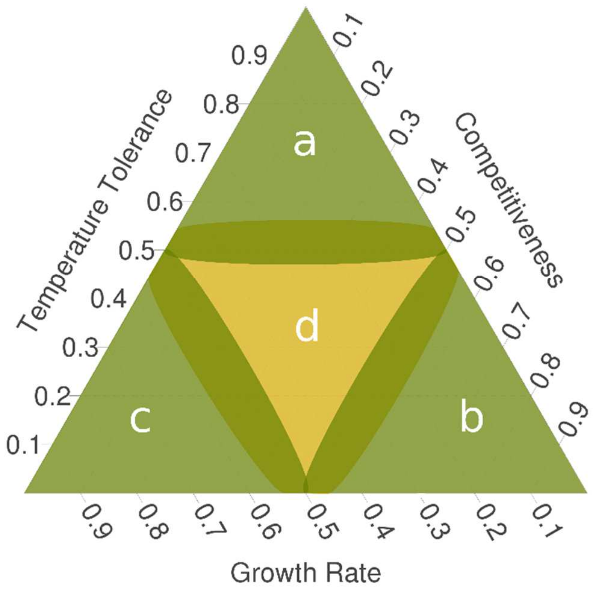

Figure S2 The classification of the coral species in dependence on their trait combinations: (a) temperature tolerant/bleaching-resistant, (b) competitive, (c) fast growing and (d) evenly distributed resources over two or all three traits.

Coral growth and competition

Spatial extension is the main trait driving the direct interaction of coral colonies, and hence if an individual colony is going to survive or perish. Each individual coral colony within the simulation grows with a species specific growth rate, assigned during the advent of the trait combinations, as described above. We used data from an extensive literature review on coral growth rates to apply natural ranges [28].

The *CI* (defined according to [29]) is a measure of aggressiveness in direct competition and ranges from 1-10 (i.e. the higher the index for a species the more successful an individual colony will be in direct competition). If the *CI* of a direct neighbor (CI_o) is higher than that of

the particular (focal) coral colony (CI_f), the initial growth rate (gR_{ini}) of the focal coral is reduced accordingly (gR_f) in the opponent's direction (Equation 1).

(1) $$gR_f = gR_{ini} \times \left(\frac{CI_f}{(CI_o \times af)}\right); CI_f \leq CI_o$$

The growth rate of a superior opponent is not affected by competition. If more than one neighbor grows into the area covered by a coral colony, their respective overlap is added up, and, if more than 75 % of a coral's covered area is occupied by other organisms the coral individual is removed from the simulation, and the previously occupied area returns to basic substrate (i.e. is available to other organisms). A branching coral is also removed if a massive coral grows over its center, where it has its base.

Coral Bleaching

Calculation of bleaching probabilities

At the end of each month, if 40 degree heating days (*DHD*) were exceeded in the last 120 days, we calculated the respective heat rate (*HR*) for this month. The maximum heat rate (HR_{max}) of 3.5 was defined according to [30] (i.e. the *HR* at which all corals bleach and die). From this value we estimated a minimum heat rate (HR_{min}) and specific minimum bleaching probabilities (bp_{min}) to fit the bleaching and mortality index for the temperature data of the year 1998 [31]. The actual bleaching probability (bp_{actual}) was then determined in direct dependence of the actual *HR* (HR_{actual}; Equation 2).

(2) $$bp_{actual} = \left(\frac{(1 - bp_{min})}{(HR_{max} - HR_{min}) \times (HR_{actual} - HR_{min})}\right) + bp_{min}$$

Corals differ in susceptibility and bleaching mortality according to their morphology, with some massive coral species being slightly more tolerant to higher temperatures [31–33].

Coral Reproduction

A stock-recruitment relationship determines how many larvae are produced in relation to the population size of a coral species. As we want to minimize variability in non-tested parameters (noise) in the model, we normalized the reproductive output for every coral species to coral surface factor with each species producing 1300 larvae cm^{-2} of their hemispherical surface area. The retention factor, which determines how many larvae will enter the system within a recruitment event, is also identical for all species. If a coral larva settles on a living organism it dies and is removed from further simulations. Branching corals can also reproduce asexually via fragmentation, either if they reach their maximum colony size, or if they are mechanically disturbed.

Macroalgae and coral-algae interactions

The interactions with algae are implemented as outlined in an earlier work [28]. Macroalgal abundances were implemented to fluctuate around 5 % coverage. They were parameterized to depict the characteristics of *Sargassum* spp. [34,35]. The growth rate of an individual was set to 30 cm per month and was reduced by 10 % if it either hit a coral or a macroalgal individual, due to the costs/consequences of competition. If taller than 30 cm in height, a macroalga had the potential to produce a fragment that was allowed, in turn, to settle within a radius of 5 m around its original thallus. If settling on a living organism, macroalga were killed and removed from the simulation. In contact with a competing macroalga the growth rate of a smaller coral colony was reduced. As above, corals were removed from the simulation area if more than 75 % of the area of an individual was covered by other organisms. Only coral

larvae interacted with turf algae, which was represented by a grid of 1 x 1 m cells. While coral recruits were ≤ 4 months old, their survival probability was reciprocal to the percentage cover of turf cells.

References

1. Edinger, E. Normal Coral Growth Rates on Dying Reefs: Are Coral Growth Rates Good Indicators of Reef Health? *Mar. Pollut. Bull.* **40,** 404–425 (2000).

2. Guzman, H. M., Cortes, J., Guzmán, H. & Cortés, J. Growth rates of eight species of scleractinian corals in the eastern Pacific (Costa Rica). *Bull. Mar. Sci.* **44,** 1186–1194 (1989).

3. Supriharyono, Indonesia, S. & Sciences, M. Growth rates of the massive coral *Porites lutea* Edward and Haime, on the coast of Bontang, East Kalimantan, Indonesia. *J. Coast. Dev.* **7,** 143–155 (2004).

4. Bessat, F. & Buigues, D. Two centuries of variation in coral growth in a massive Porites colony from Moorea (French Polynesia): a response of ocean-atmosphere variability from south central Pacific. *Palaeogeogr. Palaeoclimatol. Palaeoecol.* **175,** 381–392 (2001).

5. Suzuki, A. *et al.* Intercolony variability of skeletal oxygen and carbon isotope signatures of cultured Porites corals: Temperature-controlled experiments. *Geochim. Cosmochim. Acta* **69,** 4453–4462 (2005).

6. Oliver, J. K. J. K., Chalker, B. E. B. E. & Dunlap, W. C. W. C. Bathymetric adaptations of reef-building corals at Davies reef, Great Barrier Reef, Australia. I. Long-term growth responses of *Acropora formosa* (Dana 1846). *J. Exp. Mar. Bio. Ecol.* **73,** 11–35 (1983).

7. Jinendradasa, S. S. & Ekaratne, S. U. K. Linear extension of *Acropora formosa* (Dana) at selected reef locations in Sri Lanka. *Proc. 9th Int. Coral Reef Symp. Bali Indones. 23-27 Oct. 2000, Vol.1* **1,** (2000).

8. Harriott, V. J. Growth of the staghorn coral *Acropora formosa* at Houtman Abrolhos, Western Australia. *Mar. Biol.* **132,** 319–325 (1998).

9. Oliver, J. K. Intra-colony Variation in the Growth of *Acropora formosa*: Extension Rates and Skeletal Structure of White (Zooxanthellae-free) and Brown-Tipped Branches. *Coral Reefs* **3,** 139–147 (1984).

10. Dennison, W. C. & Barnes, D. J. Effect of water motion on coral photosynthesis calcification. *J. Exp.*

Mar. Bio. Ecol. **115,** 67–71 (1988).

11. Charuchinda, M., Hylleberg, J., Reefs, C., Charuchinda, M. & Hylleberg, J. Skeletal Extension of *Acropora formosa* at a Fringing Reef in the Andaman Sea. *Coral Reefs* **3,** 215–219 (1984).

12. Wellington, G. M. An experimental analysis of the effects of light and zooplankton on coral zonation. *Oecologia* **52,** 311–320 (1982).

13. Grigg, R. W. Depth limit for reef building corals in the Au'au Channel, S.E. Hawaii. *Coral Reefs* **25,** 77–84 (2006).

14. Neudecker, S. Growth and Survival of Scleractinian Corals Exposed to Thermal Effluents at Guam. in *Proceedings of the 4th International Coral Reef Symposium* 173–180 (1981). at <http://agris.fao.org/agris-search/search/display.do?f=1985/XB/XB85038.xml;XB8311021>

15. Harriott, V. J. Coral growth in subtropical eastern Australia. *Coral Reefs* **18,** 281–291 (1999).

16. Weil, S. M., Buddemeier, R. W., Smith, S. V & Kroopnick, P. M. The stable isotopic composition of coral skeletons: control by environmental variables. *Geochim. Cosmochim. Acta* **45,** 1147–1153 (1981).

17. Romano, S. Long-term effects of interspecific aggression on growth of the reef-building corals *Cyphastrea ocellina* (Dana) and *Pocillopora damicornis* (Linnaeus). *J. Exp. Mar. Bio. Ecol.* **140,** 135–146 (1990).

18. Glynn, P. W. & Stewart, R. H. Distribution of Coral Reefs in the Pearl Islands (Gulf of Panama) in Relation to thermal conditions. *Limnol. Oceanogr.* **18,** 367–379 (1973).

19. Le Tissier, M. D. A. The growth and formation of branch tips of *Pocillopora damicornis* (Linnaeus). *J. Exp. Mar. Bio. Ecol.* **124,** 115–131 (1988).

20. Ward, S. The effect of damage on the growth, reproduction and storage of lipids in the scleractinian coral *Pocillopora damicornis* (Linnaeus). *J. Exp. Mar. Bio. Ecol.* **187,** 193–206 (1995).

21. de Villiers, S., Shen, G. T. & Nelson, B. K. The Sr/Ca-temperature relationship in coralline aragonite: Influence of variability in (Sr/Ca). *Science (80-.).* **58,** 197–208 (1994).

22. Grottoli, A. G. Variability of stable isotopes and maximum linear extension in reef-coral skeletons at Kaneohe Bay, Hawaii. *Mar. Biol.* **135,** 437–449 (1999).

23. Smith, L. W., Barshis, D. & Birkeland, C. Phenotypic plasticity for skeletal growth, density and

calcification of *Porites lobata* in response to habitat type. *Coral Reefs* **26,** 559–567 (2007).

24. Floral, C. J. & Ely, P. S. Surface Growth Rings of *Porites lutea* Microatolls Accurately Track Their Annual Growth. *Nonhwest Sci.* **77,** (2003).

25. Rosenfeld, M., Yam, R., Shemesh, A. & Loya, Y. Implication of water depth on stable isotope composition and skeletal density banding patterns in a *Porites lutea* colony: results from a long-term translocation experiment. *Coral Reefs* **22,** 337–345 (2003).

26. Highsmith, R. Coral growth rates and environmental control of density banding. *J. Exp. Mar. Bio. Ecol.* **37,** 105–125 (1979).

27. Tanzil, J. T. I., Brown, B. E., Tudhope, a. W. & Dunne, R. P. Decline in skeletal growth of the coral *Porites lutea* from the Andaman Sea, South Thailand between 1984 and 2005. *Coral Reefs* **28,** 519–528 (2009).

28. Kubicek, A., Muhando, C. & Reuter, H. Simulations of long-term community dynamics in coral reefs - how perturbations shape trajectories. *PLoS Comput. Biol.* **8,** e1002791 (2012).

29. Langmead, O. & Sheppard, C. Coral reef community dynamics and disturbance: a simulation model. *Ecol. Modell.* **175,** 271–290 (2004).

30. Maynard, J. a. *et al.* ReefTemp: An interactive monitoring system for coral bleaching using high-resolution SST and improved stress predictors. *Geophys. Res. Lett.* **35,** 1–5 (2008).

31. McClanahan, T. R. The relationship between bleaching and mortality of common corals. *Mar. Biol.* **144,** 1239–1245 (2004).

32. Hoegh Guldberg, O. & Salvat, B. Periodic mass-bleaching and elevated sea temperatures: Bleaching of outer reef slope communities in Moorea, French Polynesia. *Mar. Ecol. Prog. Ser.* **121,** 181–190 (1995).

33. Loya, Y., Sakai, K., Nakano, Y. & Woesik, R. Van. Coral bleaching: the winners and the losers. *Ecol. Lett.* **4,** 122–131 (2001).

34. Ateweberhan, M., Bruggemann, J. & Breeman, A. Seasonal dynamics of *Sargassum ilicifolium* (Phaeophyta) on a shallow reef flat in the southern Red Sea (Eritrea). *Mar. Ecol. Prog. Ser.* **292,** 159–171 (2005).

35. Ateweberhan, M., Bruggemann, J. H. & Breeman, A. M. Seasonal Changes in Size Structure of

Sargassum and Turbinaria Populations (Phaeophyceae) on Tropical Reef Flats in the Southern Red Sea. *J. Phycol.* **45,** 69–80 (2009).
